# *Carica Papaya* leaf-infused metal oxide nanocomposite: a green approach towards water treatment and antibacterial applications

**DOI:** 10.1007/s10653-024-02090-4

**Published:** 2024-07-26

**Authors:** Rangayasami Aswini, Kannupaiyan Jothimani, Karthik Kannan, Ramyakrishna Pothu, Paramasivam Shanmugam, Rajender Boddula, Ahmed Bahgat Radwan, Govindasami Periyasami, Perumal Karthikeyan, Noora Al-Qahtani

**Affiliations:** 1 Department of Botany, Padmavani Arts and Science College for Women, Tamil Nadu, Salem, 636 011 India; 2 Research Centre for Genetic Engineering BRIN, KST soekarno JI Raya Bogor Km. 46, Cibinong, 16911 Indonesia; 3 Institute of Agricultural Engineering, Saveetha School of Engineering, Saveetha Institute of Medical and Technical Sciences, Chennai, 602105 India; 4 School of Physics and Electronics, College of Chemistry and Chemical Engineering, Hunan University, Changsha, 410082 People’s Republic of China; 5 Department of Chemistry, Faculty of Science and Technology, Thammasat University, Pathum Thani, 12120 Thailand; 6 Center for Advanced Materials (CAM), Qatar University, 2713 Doha, Qatar; 7 Department of Chemistry, College of Science, King Saud University, P.O. Box 2455, Riyadh, 11451 Saudi Arabia; 8 Department of Chemistry and Biochemistry, Ohio State University, 151 Woodruff Avenue, Columbus, OH 170A CBEC43210 USA; 9 Central Laboratories Unit (CLU), Qatar University, 2713 Doha, Qatar

**Keywords:** Green synthesis, ZnO–CuO nanocomposites, *Carica papaya* leaf extract, Photodegradation, Antibacterial activity

## Abstract

This study successfully synthesized ZnO–CuO nanocomposite using the hydrothermal method with *Carica papaya* leaf extract. The incorporation of the leaf extract significantly enhanced the nanocomposite properties, a novel approach in scientific research. Characterization techniques, including X-ray diffraction, Fourier Transmission Infrared spectroscopy, and Scanning Electron Microscopy with Energy Dispersive X-Ray Analysis, confirmed a cubic crystal structure with an average size of 22.37 nm. The Fourier Transmission Infrared spectrum revealed distinctive vibrations at 627, 661, and 751 cm^−1^ corresponding to ZnO–CuO nanocomposite corresponding to stretching and vibration modes. SEM images confirmed a cubic-like and irregular structure. The nanocomposite exhibited outstanding photocatalytic activity, degrading methylene blue dye by 96.73% within 120 min under visible light. Additionally, they showed significant antimicrobial activity, *inhibiting Staphylococcus aureus* (20 mm) and *Klebsiella pneumonia* (17 mm). The results highlight the efficiency of *Carica papaya* leaf-derived ZnO–CuO nanocomposite for environmental and health challenges.

**Graphical abstract:**

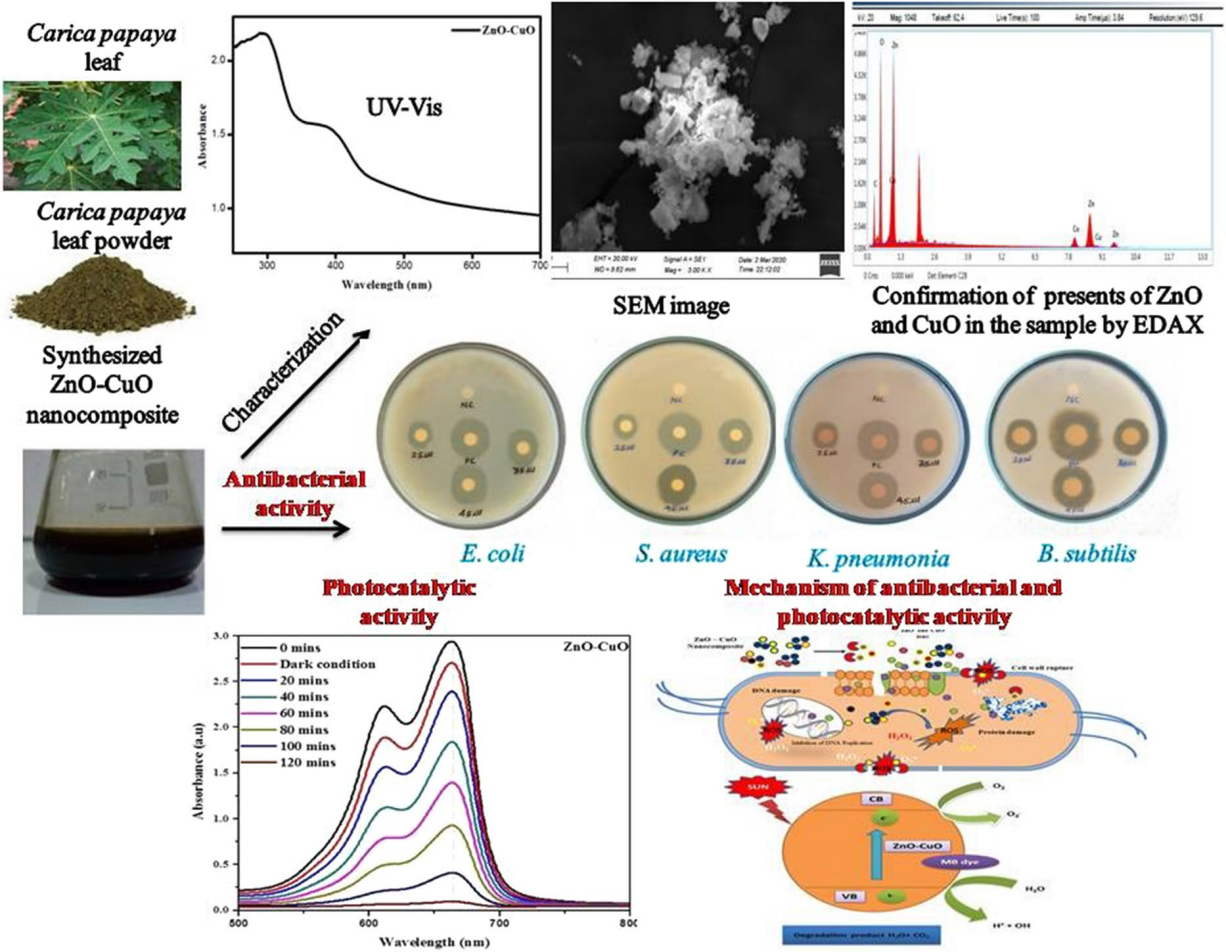

## Introduction

Research in the subject of nanotechnology is receiving significant attention globally in recent times. Many nanoparticles have potential uses in the culinary, pharmaceutical, medical, water treatment, and cosmetic industries due to their unique shape and crystallite size (Ghitman et al., 2020; Shanmugam et al., 2024; Usan et al., 2024). The metal nanoparticles offer a wide range of potential uses in industries such as photonics, electronics, catalysis, and the treatment of industrial and medical wastewater (TaghaviFardood et al., 2020). Several reports on the special qualities and practical uses of metal oxide nanocomposites made of gold, silver, palladium, iron, platinum, zinc, titanium, and other elements (Mahmoud et al., 2021). Metal oxide nanocomposite has shown incredible rest in various scientific disciplines. Among them, copper oxide (CuO) and zinc oxide (ZnO) nanocomposite garnered significant attention due to their unique properties (Akmaz et al., 2013; Ngullie et al., 2022). Zinc oxide (ZnO) nanoparticles are a type of nanoparticle that is biocompatible, multifunctional, and non-toxic. They are used in a wide range of industries, including sunscreens, antimicrobial agents used in food, coatings, paints, optics, and the removal of pollutants from aqueous media. Finally, they are used in medical fields, including drug delivery, gene delivery, biological activity, nanomedicine, and nanodiagnostics (Beura et al., 2021; Bocca et al., 2018; Kamburova et al., 2021; Rajivgandhi et al., 2021). ZnO nanostructures' high surface-to-volume ratio contributes to their appealing chemical and physical characteristics. Researchers have been paying close attention to ZnO because of its high catalytic activity, availability, stability, and biocompatibility. It is important to note that the increased surface area of ZnO nanostructure makes it more effective at photocatalysis and antibacterial activity than ZnOmicrosized particles (Beura et al., 2021; Kaviyarasu et al., 2017; Padmavathy & Vijayaraghavan, 2008). Since it is the foundation of various high temperature superconductors and giant magneto-resistance materials, as well as a p-type semiconductor with a tight band gap (1.2 eV in bulk), cupric oxide (CuO) has been the subject of intense study attention among transition metal oxides (MOs) (Anandan & Yang, 2007; Filipic & Cvelbar, 2012). Due to their potential for use in a wide range of applications, such as gas and biosensors, photodetectors and solar cells, nanofluids, supercapacitors, catalysis, near-infrared filters, magnetic storage media, and the preparation of different organic–inorganic nanocomposite, CuO nanostructures have been thoroughly studied (Jelani et al., 2018; Zhang et al., 2014). Since CuO nanoparticles exhibit antibacterial, antifungal, and antioxidant properties, they have found extensive usage in medicine. According to a recent paper, CuO nanoparticles target Methicillin resistant *Staphylococcus aureus* and *E. Coli* biofilms and stop the growth of infections in aquaculture (Chari et al., 2017). ZnO and CuO nanocomposite stood out for their distinct characteristics, including their applications in photoconductivity, photothermal processes, electronics, optics, nanofluid technology, and photonics. Copper and zinc oxide nanoparticles (CuO and ZnO NPs) have been of extensive interest because of the part of these nanoparticles in catalysis, biofuels, and wastewater treatment, environmental and biomedical applications (Bonthula et al., 2023; Farooq et al., 2019; Gayathri et al., 2021; Lu et al., 2017; Rajender et al., 2023; Ramyakrishna et al., 2022; Shanmugam et al., 2023). Researchers are increasingly turning to the green synthesis technique due to its use of less harmful chemicals, its eco-friendly nature, and its ability to facilitate one-stage synthesis of nanoparticles (Sundrarajan et al., 2015). Biosynthesis and environmental technologies are being employed to produceCuO and ZnONPs, which are considered to be non-toxic, environmentally safe, and biocompatible. A wide range of methods is available for the green synthesis of ZnO NPs (Lakshmeesha et al., 2014). In a study conducted by Jan et al. (2019) ZnO–CuO nanocomposite were synthesized using the hydrothermal method, achieving a remarkable 56% reduction in MB dye under UV–Vis illumination. Green synthesis is the process of creating nanoparticles by using natural substances as reducing or capping agents, such as plant extracts or biodegradable materials. This approach is centered on sustainability and mitigating the ecological consequences of nanoparticle manufacturing. In hydrothermal synthesis, these can include metal salts, organic compounds, or surfactants that break down or react under hydrothermal conditions to form nanoparticles. In green synthesis, reducing agents like polyphenols, flavonoids, terpenoids, and organic acids act as reducing agents to convert metal ions into nanoparticles. High pressure and temperature are used in an aqueous solution during hydrothermal synthesis to promote the development and production of nanoparticles. The synthesis takes place in a closed container known as a hydrothermal reactor or autoclave. The hydrothermal synthesis process may be made more environmentally friendly and sustainable by using green synthesis principles, such as the use of natural reducing agents or capping agents made from plant extracts or biodegradable materials. This technology decreases the environmental effect of classic hydrothermal synthesis methods and reduces dependency on chemical reducing agents. Through the integration of green synthesis principles with hydrothermal synthesis, scientists may generate functionalized nanoparticles with customized surface chemistries, rendering them appropriate for environmental remediation, sensing, and catalytic applications. Ardekani et al. (2018) reported that N-doped ZnO–CuO nanocomposite, prepared through ultrasonic spray pyrolysis, exhibited an impressive 80% degradation rate against MO dye. Rajith Kumar et al. (2020) described the combustion method to synthesize ZnO–CuO nanocomposite, effectively reducing MB dye. Govindasamy et al. (2021a) conducted a study on ZnO–CuO nanocomposite synthesized using a green approach, which demonstrated the inhibition of the growth of gram-positive and gram-negative bacteria, *E. coli and S. aureus*. Additionally, Taufiqueet et al. (2018) reported that chemically prepared ZnO–CuO nanocomposite exhibited strong antibacterial activity against *S. aureus*.The green synthesis of CuO, ZnO, and CuO/ZnO nanocomposite for use in biological applications using plant extracts is covered in a number of studies. ZnO NPs, CuO NPs, and CuO/ZnO NCs have been produced from a variety of plant extracts based on prior literature studies. It has been reported on the green production of CuO nanoparticles utilizing *Syzygiumguineense* (SyG) leaf extract on bacteria and the assessment of their electrochemical characteristics. The CuO/ZnO nanocomposite, which lowers the band gap energy and increases particle size, may be created by adding CuO to ZnO (Qamar et al., 2015). *Penicilliumcorylophilum* strain, a species of fungus, was combined with copper and zinc precursors to biosynthesize CuO/ZnO nanocomposite for photocatalytic activity. The ZnO and CuO nanoparticles are not as stable as the produced CuO/ZnO nanocomposite (Fouda et al., 2020a). The plant's biological uses have become excessive because of the existence of extremely powerful and therapeutic bioactive components. However, the present study to synthesis of novel nanocomposite in ZnO–CuO using an aqueous extract from *Carica papaya* leaf extracts and to reports its antibacterial activity against both human and food pathogens and photocatalytic activity against organic dye.

## Materials and methods

### Materials

The plant material of *Carica papaya* was composed from house garden, Junction, Salem, Tamil Nadu. The Sigma Aldrich chemicals (analytical grade) zinc acetate (Zn(CH_3_COO)_2_.2H_2_O; 99%) and copper acetate (Cu(CH_3_COO)_2_; 99%) were brought from Progen lab chemicals, Salem.

### Synthesis of ZnO–CuO nanocomposite

**Scheme 1:**
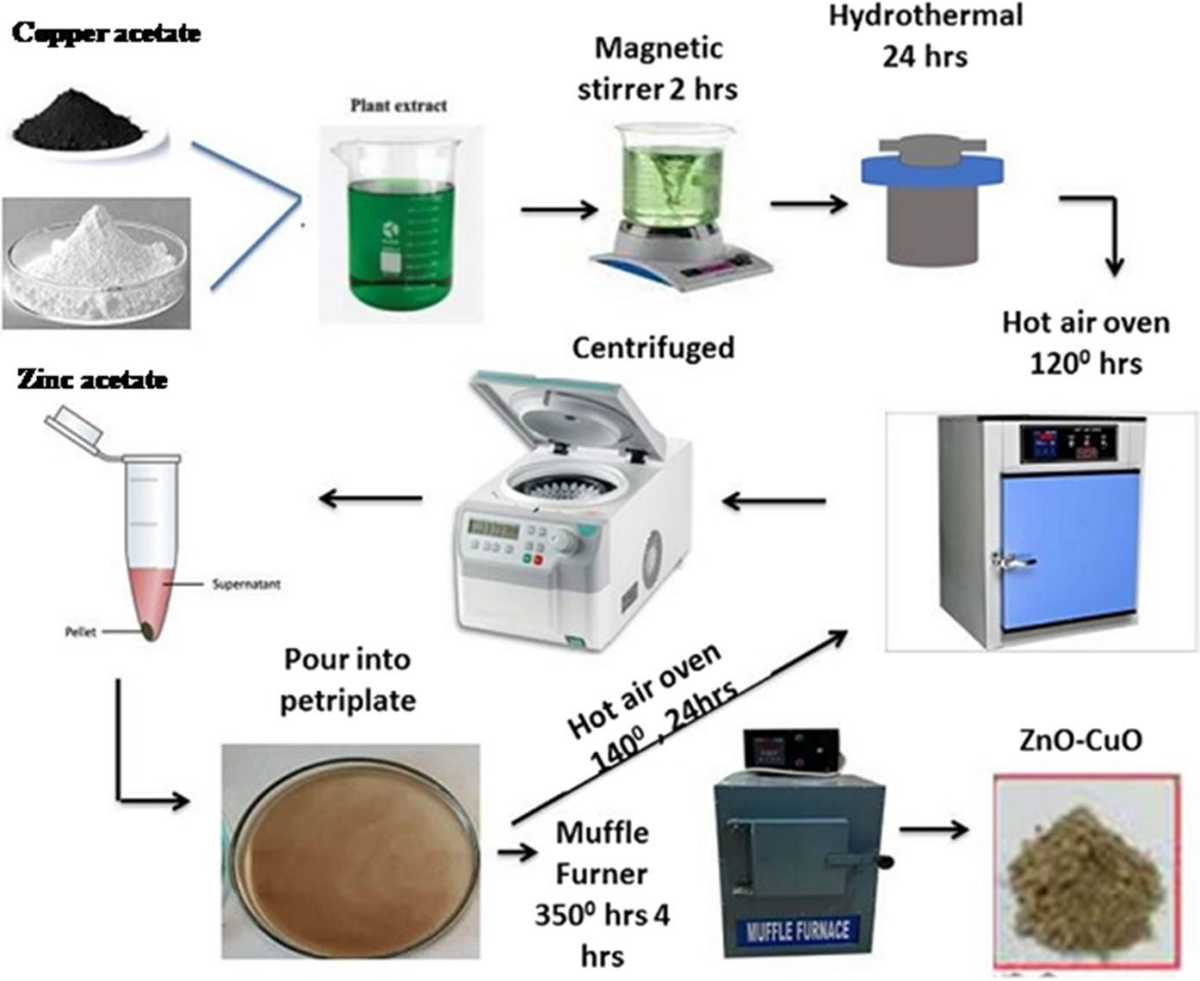
Synthesis of ZnO–CuO nanocomposite via green route

The stepwise procedure for the synthesis of biogenic ZnO and CuO nanocomposite is illustrated in Scheme 1. Synthesis of ZnO–CuO nanocomposite was followed slightly modified method by Bekru et al. (2022) was reported that, 5 g zinc acetate dissolved in 20 ml of double distilled water (DDW) and added with 20 ml of soxhlet extractor of *Carica papaya* leaf extract solution is a sample-A. In a 5 g copper acetate dissolved in 20 ml of DDW and added with 20 ml of leaf extract solution and it was marked as a sample-B. In a sample, A and B are mixed thoroughly and kept into magnetic stirring for 2 h at room temperature. After that sample was kept in hot air oven at 120 °C for 24 h. Then it was centrifuged at 5000 rpm for 15 min. In a supernatant and pellet are also separated in centrifuge tube after that supernatant is removed and are collected the pellet to pour into a petri dish. The poured petri dish was kept in a hot air oven at 24 h at 140 °C. Dried sample was collected, and it was transferred into cruciferous and using set a muffle furnace at 4 h at 350 °C. The phytochemicals found in the papaya leaf extract from *Carica*, including organic acids, polyphenols, flavonoids, and alkaloids, function as reducing agents to stabilize the CuO and ZnO nanoparticles during production. The mixture can be separated, cleaned, and dried for additional characterization after being heated to a certain temperature for a predetermined amount of time. This allows the reduction of Cu(II) ions to CuO nanoparticles and the same via the reduction of Zn(II) ions to ZnO nanoparticles.

Zn^2+^  + phytochemicals → ZnO + Reduced organic compounds.

### Instrumentation studies

The diffraction pattern of the nanocomposite was recorded by the X-ray diffractometer (Rigaku Mini Flexll desktop) in 2*θ* range 10°–80° range. Morphological and elemental analysis was carried out by scanning electron microscope (SEM-Hitachi S3400n) with EDAX. Molecular structure and functional groups were studied by FTIR (Bruker alpha-p) with the spectrum range from 400–4000 cm^−1^. The optical properties were studied by Perkin Elmer Lambda 25 spectroscopy with absorption range from 200 – 800 nm.

### Photocatalytic activity

The photocatalytic activity of ZnO–CuO nanocomposite was analyzed under solar light by evaluating the degradation of MB. About 20 mg of synthesized ZnO–CuO nanocomposite was mixed with 100 mL of MB aqueous solution. The mixture was stirred for 30 min. in dark conditions before irradiation. From that point forward, the suspension was presented to daylight illuminations. At various time stretches, barely any aliquots of color suspension were taken and centrifuged at 10,000 rpm for 10 min. and afterward absorbance range was estimated by UV-apparent spectroscopy (Liang et al., 2018). The level of photocatalytic debasement was determined from the following equation.

1 $${\text{Percentage of degradation }}\left( \% \right) \, = { 1}00 \, \left( {{\text{C}}_{0} - {\text{ C}}_{{\text{t}}} } \right) \, /{\text{C}}_{0}$$

where C_0_—initial concentration, C_t_—concentration at time (t).

### Antibacterial activity assay

Synthesized ZnO–CuO nanocomposite was analysed against Gram + ve and Gram–vebacteria through agar disc diffusion method for antibacterial activity. Clinical isolated *E. coli, S. aureus, K. pneumonia,* and *B. subtilis* were sub-cultured used by nutrient broth at 24 h 37 °C. The different concentrations (25 µL, 35 µL, and 45 µL) of ZnO–CuO nanocompositeextract from the stock solution (mg/mL). The positive and negative controls were used in this study. Kanamycin used as a positive control. The plates were kept into hot air oven at 24 h for 37 °C. The zone of inhibition (ZOI) was measured after 24 h (Rajith Kumar et al., 2020).

## Results and discussion

### Synthesis of ZnO–CuO nanocomposite from Carica papaya

**Fig. 1:**
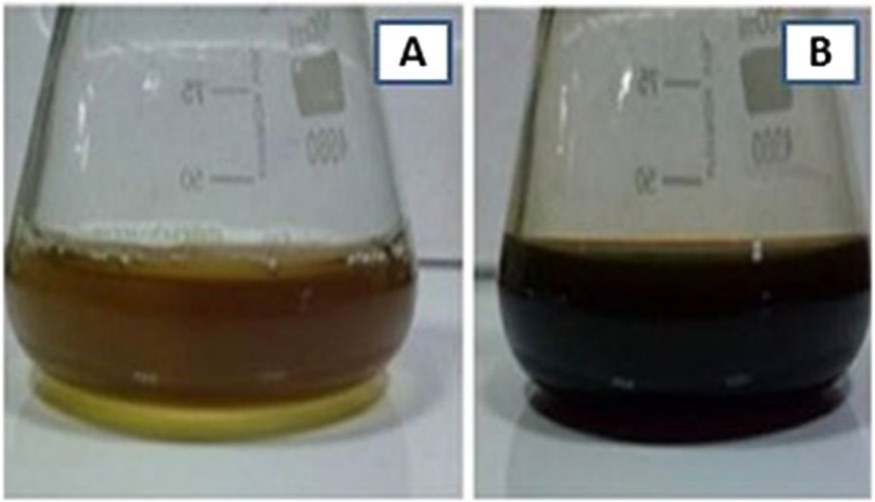
**A** Plant extract with ZnO–CuO nanocomposite, **B** Brown colour changed after 2 h

The present study, a biosynthesis of nanocomposite was prepared by using leaves of *Carica papaya* extract collected from home garden. The reduction of ZnO and CuO nanoparticles has been confirmed by visually manifested from the colour change after 24 h from green to dark brown (Fig. 1). The colour changes have been confirmed that the formation of ZnO–CuO nanocomposite. The synthesis of nanocomposite was confirmed by the observation of colour change. The CuO nanoparticles colour changed from yellow to dark green. And the synthesized ZnO nanoparticles colour changed from yellow to white. The synthesized ZnO–CuO nanocomposite colour changes to dark green (Jerry & Adeyemi. et al., 2022). The extract's bioactive components function as reducing agents, causing the Zn^2+^ and Cu^2+^ metal ions to be reduced to their corresponding metallic forms. The *Carica papaya* leaf extract contains phytochemicals that have two functions: they stabilize and reduce the freshly generated metal nanoparticles, so averting their uncontrollable agglomeration. In the extract's water, the reduced metal ions may first form metal hydroxides (Metal-OH). By stabilizing these metal hydroxides, the phytochemicals help to provide a regulated environment for further reactions. However, ZnO's absence of p-type electrical conductivity highlights how crucial it is to research hybrid heterojunctions. CuO is one of the better p-type materials to form p-n heterojunctions with ZnO, which has a wide range of applications in optoelectronic devices. CuO's nontoxicity, inexpensive market pricing, and high absorption coefficient are its primary benefits. In addition, there are numerous ways to prepare CuO, such as chemical bath deposition, thermal oxidation, magnetron sputtering, vacuum arc plasma evaporation, atomic layer deposition, electro deposition, ion sputtering, wet-chemical oxidation process, and spray pyrolysis. For use in photovoltaic systems, where CuO is used as an absorbing layer and wide band gap ZnO is used as a window layer, the combination of p-type CuO and n-type ZnO seems promising. Many kinds of gases, including CO, H_2_, volatile organic compounds, and H_2_S, have been effectively detected using CuO/ZnO junctions (Das & S. Vimal Chandra Srivastava. 2018; Yatskiv et al., 2019).

### Structural studies

**Fig. 2:**
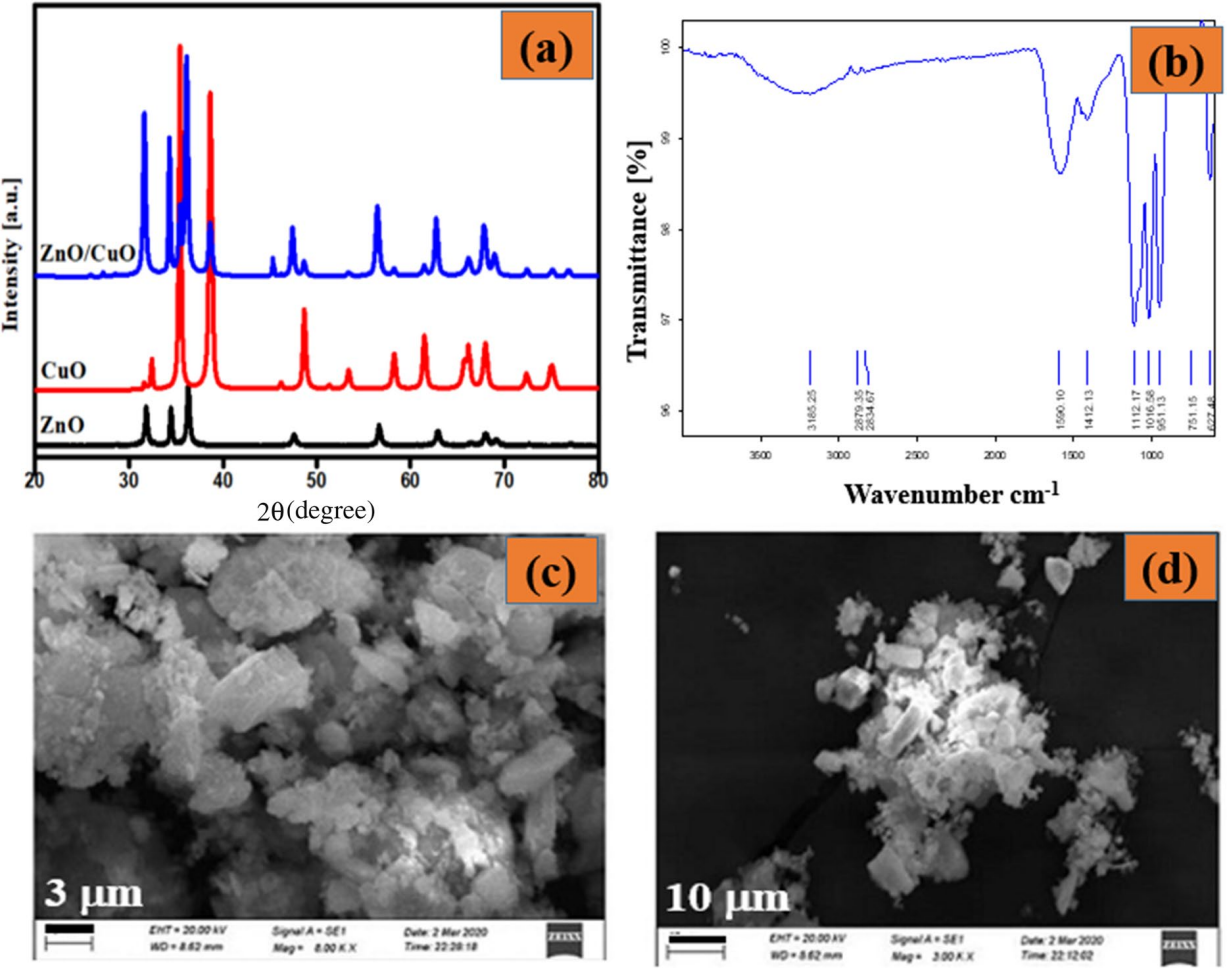
**a** XRD pattern of synthesized ZnO, CuO and ZnO–CuO nanocomposite., **b** FTIR spectrum of ZnO–CuO composites, **c-d** SEM images of ZnO–CuO nanocomposite with different magnifications

The crystallite size and structure of the as-prepared ZnO–CuO nanocomposite were characterized by XRD techniques. The XRD pattern of biosynthesized ZnO, CuO and ZnO–CuO nanocomposite from *Carica papaya* is show in Fig. 2a.The X-ray diffraction patterns of ZnO at 30.98°, 34.22°, 36.41°, 46.602°, 57.64°, 63.01°, 66.48°, and 67.97° corresponds to the (100), (002), (101), (102), (110), (103), (200), and (112) planes, respectively. These crystalline reflection data exhibit excellent agreement with the standard JCPDS 36-1451. Additionally, the observed diffraction peaks of CuO at 36.26, 37.94, 48.33, 54.13, and 59.01 values align well with the corresponding data values in the JCPDS 89-5895 dataset. Further, XRD pattern of ZnO/CuO composites materials containing all the diffraction peaks of ZnO and CuO. Thus, peak evidence confirmed the formation of ZnO/CuO nanocomposites (Klinbumrung et al., 2014). ZnO–CuO nanocomposite exhibited both phases of hexagonal and monoclinic structure of ZnO and CuO (Li & Wang, 2010; Yulizar et al., 2018). Moreover, the average crystalline size was calculated major intensity peaks using the Scherrer's equation. The *2θ*, FWHM, and particle size values are given in the Table 1.

**Table 1:** XRD data (2θ position, FWHM and particle size

Sample	*2θ*	FWHM	Particle size
ZnO	36.32	0.3979	21.95
CuO	38.64	0.3219	27.33
ZnO/CuO	36.32	0.3903	22.37

### FTIR study

The surface functional groups, and formation of ZnO–CuO nanocomposite were confirmed by FTIR analysis and the corresponding spectra shown in Fig. 2b. The FTIR spectrum of *Carica papaya* extract exhibits peaks at 3185.25, 2879.35, 2834.67, 1590.10, 1412.13, 1112.17, 1016.58, and 951.13 cm^−1^. The robust absorption bands at 3185.25 and 2879.35 cm^−1^ are associated with the bending vibrations of C–H stretching groups in alkynes and H–C–H alkanes. Further, bands at 2834.67 and 1590 cm^−1^ are attributed to the H–C–H and N–H bending groups of amides, respectively. Moreover, the peaks at 1412.13 and 1112.17 cm^−1^ can be linked to the C–C=C asymmetric stretch group in aromatic rings and the aliphatic group in ethers. The band at 1016.58 cm^−1^ is assigned to the C=N stretching vibration of aliphatic amines (Bordbar et al., 2018; Preethi et al., 2020). The plant extract included polyphenolic chemicals, primarily phenolic acids, which functioned as both stabilizing and reducing agents to produce ZnO/Fe_3_O_4_ NCs, according to Fourier transform infrared (FTIR) spectroscopy data (Dwivedi et al., 2021; Govindasamy et al., 2021b). Furthermore, the peak observed at 627 cm^−1^ is indicative of Zn–O and Cu–O stretching vibrations. This peak evidence confirmed the formation of ZnO–CuO nanocomposites. Moreover, all the FTIR spectral evidence supports the plant extract was successfully coated onto the surface of ZnO–CuO nanocomposites.

### Morphological studies

**Fig. 3:**
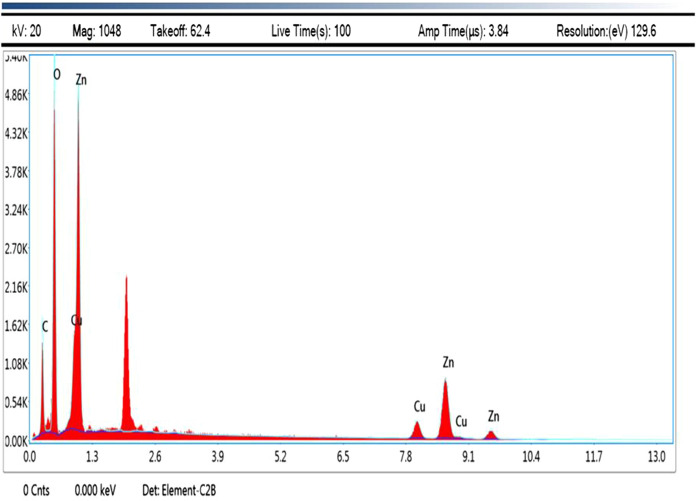
EDAX spectrum of ZnO–CuOnanocomposite (Inset: Inside EDAX area marked)

The surface structure of the nanocomposite was examined using SEM analysis (Fig. 2c, d).Its show the cubic and irregular shape of the nanocomposite. FEDAX analysis exhibits the presence of Cu, Zn, and O that’s proving the purity of the biosynthesized nanocomposite(Fig. 3). It was revealed of Zn (39.15%), Cu (19.82%), and O (32.99%). No impurities peaks were found, and it indicates the purity of biosynthesized nanocomposite. The present results were overlapped with previously reported work (Rajith Kumar et al., 2020).

### Optical studies

**Fig. 4:**
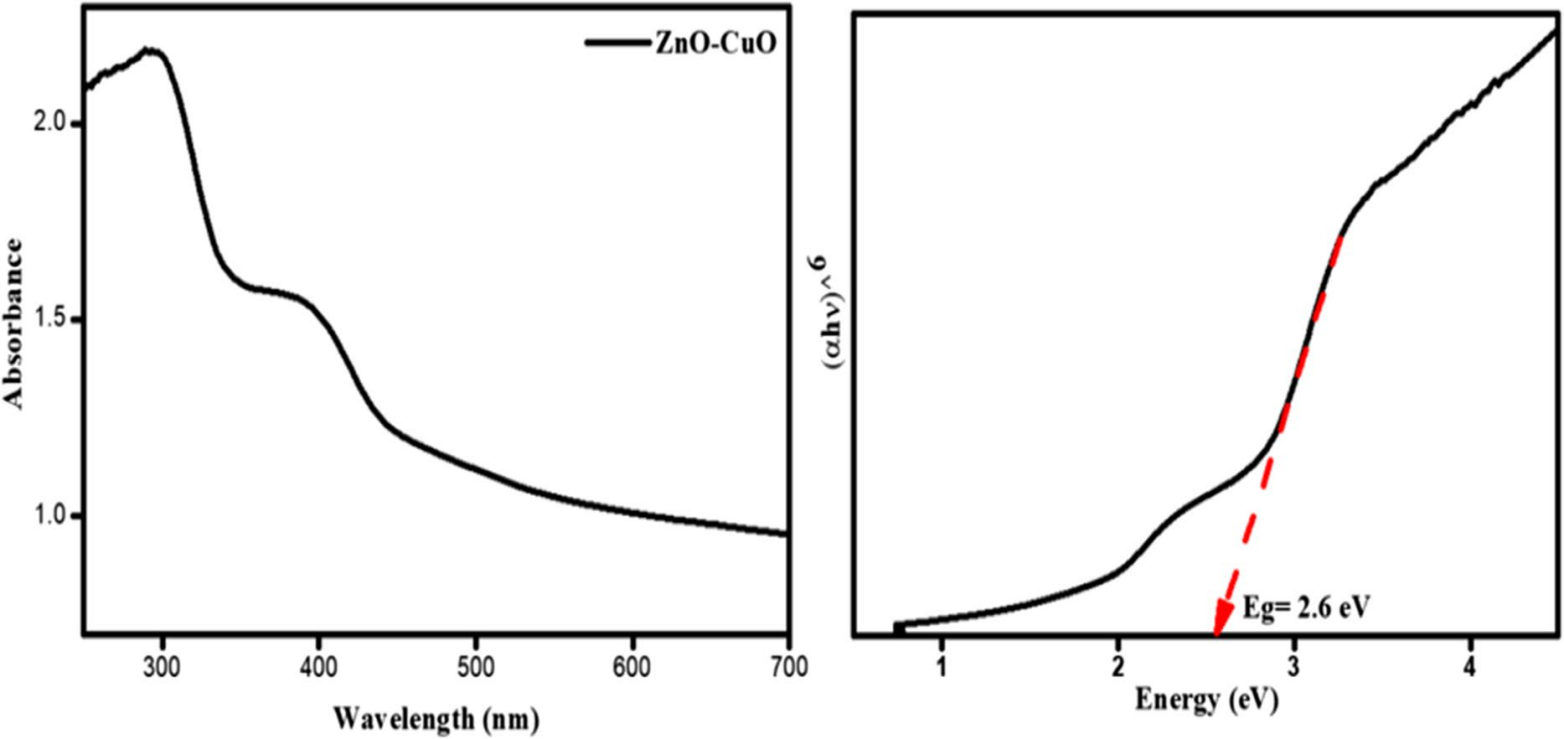
Diffuse reflectance spectroscopy (DRS-UV) and Tauc plot for the ZnO/CuO composites

The optical properties of the prepared ZnO–CuO nanocomposite were analyzed by DRS UV–Vis spectroscopy, and the corresponding images are shown in Fig. 4a. It can be observed that the prepared ZnO–CuO nanocomposites exhibit absorbance in the range from 300 to 450 nm. The ZnO–CuO nanocomposites show observed peaks that confirm the presence of ZnO and CuO (Koshimitsu et al., 2020). The observed peaks above 400 nm indicate that the prepared ZnO–CuO nanocomposites absorb visible light. The absorption of visible light favours the improvement of photocatalytic activity (Mohammadi-Aloucheh et al., 2018). Furthermore, based on the DRS UV–Visible spectrum, the energy band gap was calculated by Tauc plot (Fig. 4b). The calculated energy band gap values of the ZnO/CuO nanocomposite are 2.6 eV. Results from UV–Vis DRS verify the feasibility of the ZnO/CuO nanocomposite in the visible region.

### Photocatalytic activity

**Fig. 5:**
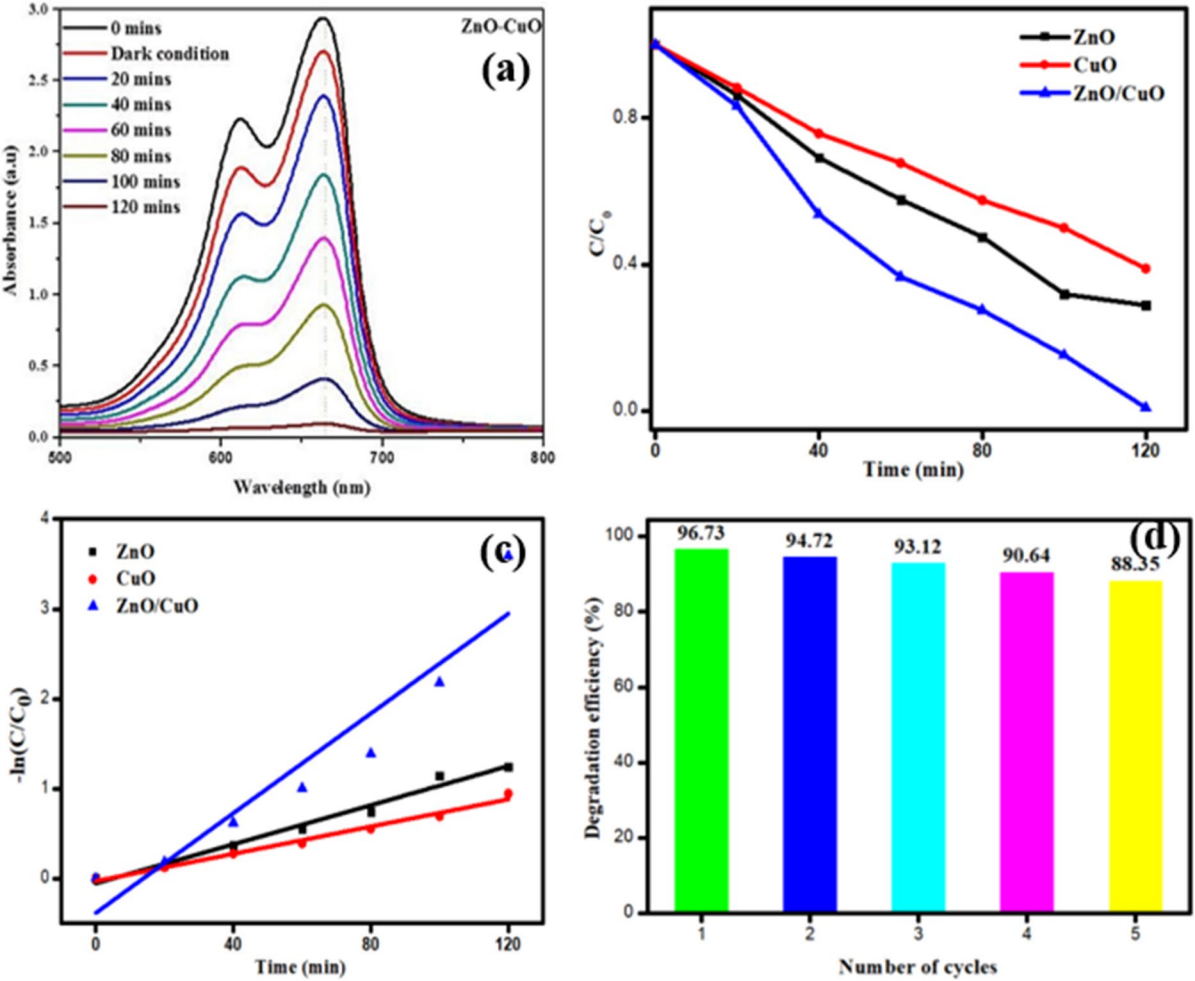
**a** UV–Vis spectra of MB over ZnO/CuO nanocomposites **b** Photocatalytic degradation efficiency of ZnO, CuO and ZnO/CuO nanocomposites, **c** Pseudo-first order kinetic plots for ZnO, CuO and ZnO/CuO nanocomposites, and **d** recycling efficiency of MB over the presence of ZnO–CuO composites

The photocatalytic action of ZnO–CuO nanocomposite was inspected for MB colour under daylight light. The debasement absorbance of MB colour was estimated diverse time spans displayed in Fig. 5a. The maximum absorption nanocomposite peaks at near 670 nm for MB was noticed. The control experiment showed that MB does not degrade in the absence of photocatalysts, indicating that MB is more stable under these conditions. The degradation efficiency of the ZnO, CuO and ZnO/CuO nanocomposites for 56.21%, 42.32% and 96.73% of MB dye degradation activity (Tables 2 and 3). On comparing ZnO, CuO and ZnO/CuO nanocomposites, the ZnO/CuO composites shows better outstanding catalytic activity than ZnO and CuO nanoparticles. The composites materials prevent the electron hole pair recombination (Balaji et al., 2023; Thomas et al., 2022).The absorbance of dye decreases while increasing the time which indicate the very good dye degradation of ZnO–CuO nanocomposite. The photocatalytic activity of ZnO–CuO nanocomposite compared with other reported metal oxide nanoparticles was mentioned in Table 2. Sakib et al. have reported that, the ZnO–CuO nanocomposite was showed 91% of dye degradation against MB dye. Ullah et al. has studied that, the Cauliflower and potatoes peal wastes used to synthesizeZnO–CuO nanocomposite by facial chemical route for photocatalytic degradation applications. Based on these results, this prepared ZnO–CuO nanocomposite having higher MB dye degradation activity (Rajith Kumar et al., 2020; Sakib et al., 2019; Ullah et al., 2020).

**Table 2:** Comparison of MB dye degradation efficiency of the prepared ZnO–CuO nanocomposite with previously reported works

Samples	k_obs_	R^2^
ZnO	0.01088	0.9915
CuO	0.00759	0.9938
ZnO/CuO	0.02779	0.9618

**Table 3:** Comparison of MB dye degradation efficiency of the prepared ZnO–CuO nanocompositewith previously reported works

Catalysts	Preparation method	Light source	Degradation time (min)	Degradation of efficiency (%)	References
CS—ZnO	Green chemistry	Sunlight	80	82	Bharathi et al., (2019)
GO—CuO	Green synthesis	Visible light	60	83.20	Ganesan et al., (2020)
Ni@Fe_3_O_4_	Green synthesis	UV light	30	89	Pakzad et al., (2019)
rGo—ZnO	Chemical	Visible light	75	57.14	Nithiyadevi and Ravichandran (2017)
ZnO-CdO-CuO	Green	Sunlight	100	94	Munawar et al., (2020)
Ce-Doped ZnO	Precipitation	UV-light	60	95	Sa-nguanprang et al., (2020)
Ni-Doped CuO	Sonication	UV-light	60	52	George et al., (2022)
Zn Doped CuO				63	
Fe Doped CuO				62	
CuOZnO	Chemical	Visible light	90	95	Lu et al., (2021)
ZnO/CuO	One step polyacrylamide gel method	UV-light	140	71	Wang et al., (2022)
ZnO – CuO	Green synthesis	Sunlight	60	96.73	Present work

The examination of the MB degradation kinetics for the prepared catalysts indicates the application of a first-order kinetic model to the experimental data. The equation − ln(C_0_/C) = kt was employed to determine the reaction rate of the sample, where C_0_ and C represents the initial and final concentration of MB, and k and t denote the rate constant and time, respectively. The linear correlation between ln(C_0_/C) and t illustrates the degradation of MB, conforming to pseudo-first-order kinetics for the prepared catalysts (Fig. 5c). The pseudo-first-order kinetic fit is evidently confirmed by the observed results. Based on the kinetic plot, rate constants (k_obs_) and correlation coefficients (R^2^) were calculated. The determined rate constants (k_obs_) and agreeing correlation coefficients (R^2^) indicate a substantial enhancement in dye degradation in the presence of ZnO–CuO nanocomposites. This suggests that the ZnO–CuO nanocomposites exhibit higher photocatalytic performance, revealing their effectiveness in preventing photocorrosion, suppress the recombination of electron hole pairs, and enhance active sites. Moreover, the recycling effectiveness of the prepared catalysts was assessed by subjecting them to the degradation of MB under consistent experimental conditions. Following each test, the catalysts were gathered using a centrifugation process, cleansed with deionized water, and subsequently dried in a hot air oven. The recycling assessment was performed over the course of five cycles under identical experimental conditions (Fig. 5d). Throughout these cycles, the degradation efficiency of each iteration remained consistent. This data indicates the commendable recycling properties of the ZnO–CuO nanocomposites.

Potential mechanism for photocatalysis might be that ZnO and CuO nanoparticles absorb light, which sets off the degradation process. Because ZnO and CuO have similar bandgap energies, they can both absorb UV–visible photons and produce electron–hole pairs (e⁻-h⁺). Electrons (e⁻) in ZnO and CuO's valence band (VB) are stimulated to the conduction band (CB) upon light absorption, forming electron–hole pairs (e⁻-h⁺). These very reactive entities, known as electron–hole pairs, are essential to the degradation process. After reacting with molecular oxygen (O₂) or water (H₂O) adsorbed on the surface of the nanoparticles, the photogenerated electrons (e⁻) in the ZnO/CuO conduction band can produce superoxide radicals (·O₂⁻) or hydroxyl radicals (·OH), respectively. These radicals are potent oxidizers that may degrade organic compounds. The ZnO/CuO nanoparticles' surface is adsorbed with the dye molecules found in the solution. The adsorption stage is crucial because it brings the reactive species (·O₂⁻, ·OH) produced during the photocatalytic process near the dye molecules. The reactive species causes oxidation processes that break down the dye molecules once they have been adsorbed onto the surface of the nanoparticle.

Specifically, the ·OH radicals are very good at rupturing the chemical bonds that hold the dye molecules together, which causes the dye to degrade into less toxic, smaller compounds. Under perfect circumstances, the degradation process keeps going until all the dye molecules have calcified into inorganic compounds, such water (H_2_O), carbon dioxide (CO₂), and other simple molecules. The full eradication of dye contaminants from the environment depends on this mineralization process. In a nanocomposite, ZnO and CuO together can have synergistic effects that increase the photocatalytic activity. As an illustration, the heterojunction between ZnO and CuO nanoparticles can help with charge transfer and separation, increasing the dye degradation process's overall efficiency.

### Antibacterial activity

**Fig. 6:**
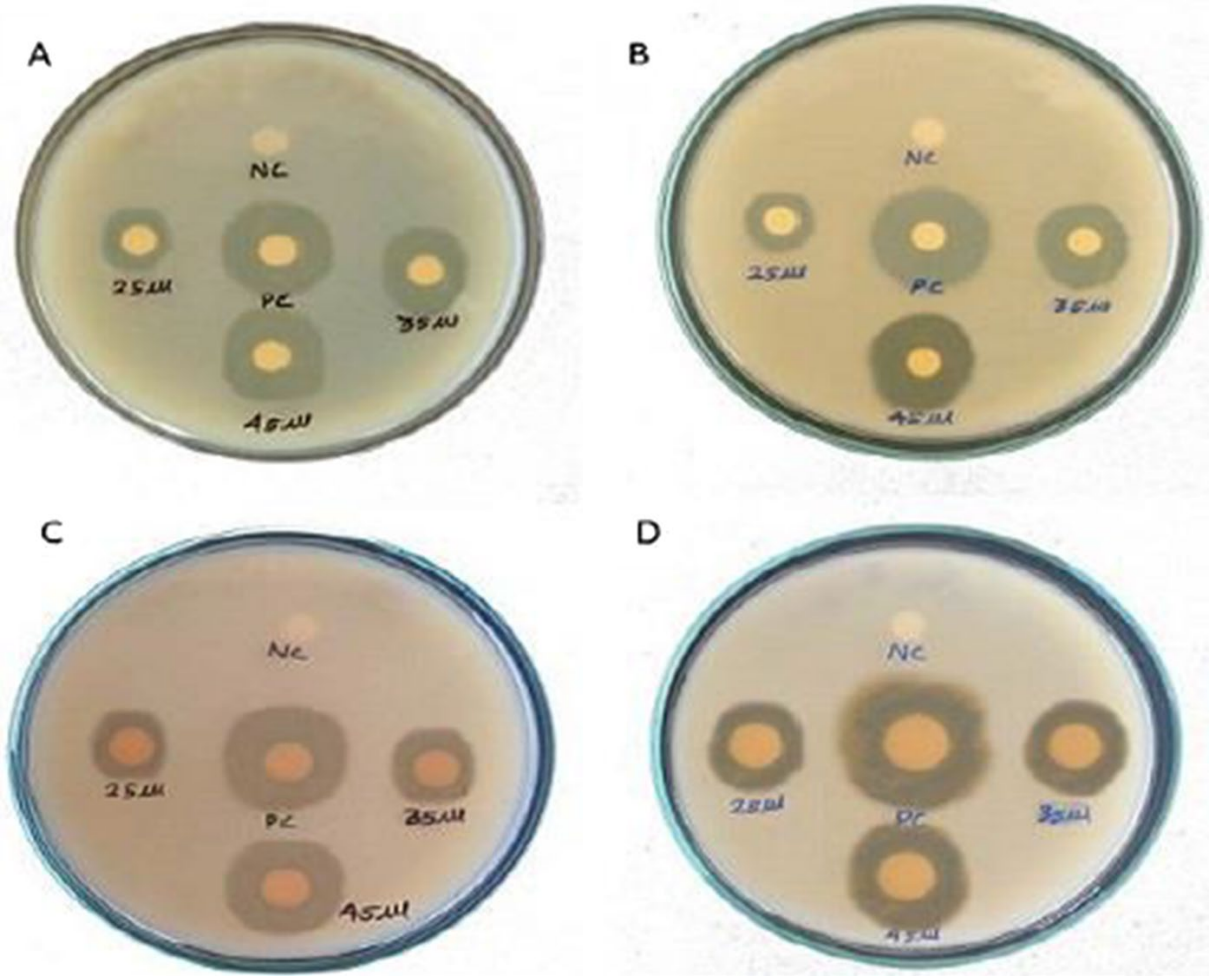
Antibacterial activity of synthesized ZnO–CuO nanocomposite*.* **A** E. coli, **B** S. aureus, **C** K. pneumonia, and **D** B. subtilis

Figure 6 shows the antibacterial activity of ZnO–CuO nanocomposite against G + ve and G-ve bacteria (*E. coli, S. aureus, K. pneumonia,* and *B. subtilis*) using disc diffusion method. The different concentrations were used from 25 to 45 µL. The nanocomposite displayed greater antibacterial activity against *S. aureus* (20 mm), *K. pneumonia* (17 mm) at a concentration 45 µL compared to positive control (Table 4). The evaluation of antibacterial movement used various metal oxides is shown in the Table 5. The antibacterial activity of ZnO–CuO nanocomposite wasshowing the high activity against *S. aureus* (Koshimitsu et al., 2020).Toenhances the meaningfulness of the antibacterial activity assessment, it is essential to conduct a dose–response analysis. This involves evaluating the antibacterial efficacy at various concentrations of the nanocomposite to identify an optimal concentration that exhibits effective antibacterial activity while minimizing toxicity. A microplate reader can be employed to assess bacterial growth inhibition or bacterial viability at different concentrations of ZnO–CuOnanocomposite. This approach allows for the generation of a dose–response curve, enabling the determination of the concentration at which antibacterial activity is maximized without causing undue harm.Moreover, defining a safe dose and understanding the potential cytotoxic effects on human cells are critical steps in assessing the applicability of these nanocomposite. It is advisable to explore the selective toxicity of the nanomaterial against bacterial cells while sparing mammalian cells. Incorporating these considerations and experimental approaches will enhance the relevance and safety assessment of the antibacterial activity of ZnO–CuO nanocomposite, providing valuable insights for their potential applications. The antibacterial activity of ZnO–CuO nanocomposite needs optimization and a dose–response analysis to determine a safe and effective concentration. Using a microplate reader, evaluating bacterial growth inhibition at various concentrations is crucial to identify the optimal antibacterial efficacy while minimizing toxicity. This approach ensures a more meaningful assessment and potential application of the nanocomposite.

**Table 4:** ZOI of the biosynthesized ZnO–CuO nanocomposite

Name of the bacteria	Gram reaction	ZOI (mm) at different concentration (µL) of ZnO–CuO nanocomposite			Negative control	Positive control
Species		25	35	45		
*E. coli*	G^−ve^	9	10	12	–	15
*S. aureus*	G^+ve^	13	15	20	–	17
*K. pneumonia*	G^−ve^	11	12	17	–	15
*B. subtilis*	G^+ve^	8	9	11	–	13

**Table 5:** Comparison of antibacterial activity with reported various metal oxides

Samples	Test of organisms	Preparation method	Concentration	ZOI (mm)	References
ZnO – CuO	*S. aureus*	Microwave	10%	17	Koshimitsu et al., (2020)
ZnO – CuO		Green synthesis	1000 µg/mL	08	Bonthula et al., (2023)
CS/ZnO		Green synthesis	20 1000 µg/mL	11	Ganesan et al., (2020)
CeO_2_-CdO	*K. pneumonia*	Chemical precipitation	200 µg/mL	12	Maria Magdalane et al., (2016)
		Hydrothermal	200 µg/mL	14	
Ag_2_O.CeO_2_.ZnO		Co precipitation	100 µL	15	Subhan et al., (2015)
ZnO–CuO		Green synthesis	45 µL	17	Present work

## Antibacterial and dye degradation possible mechanism

**Fig. 7:**
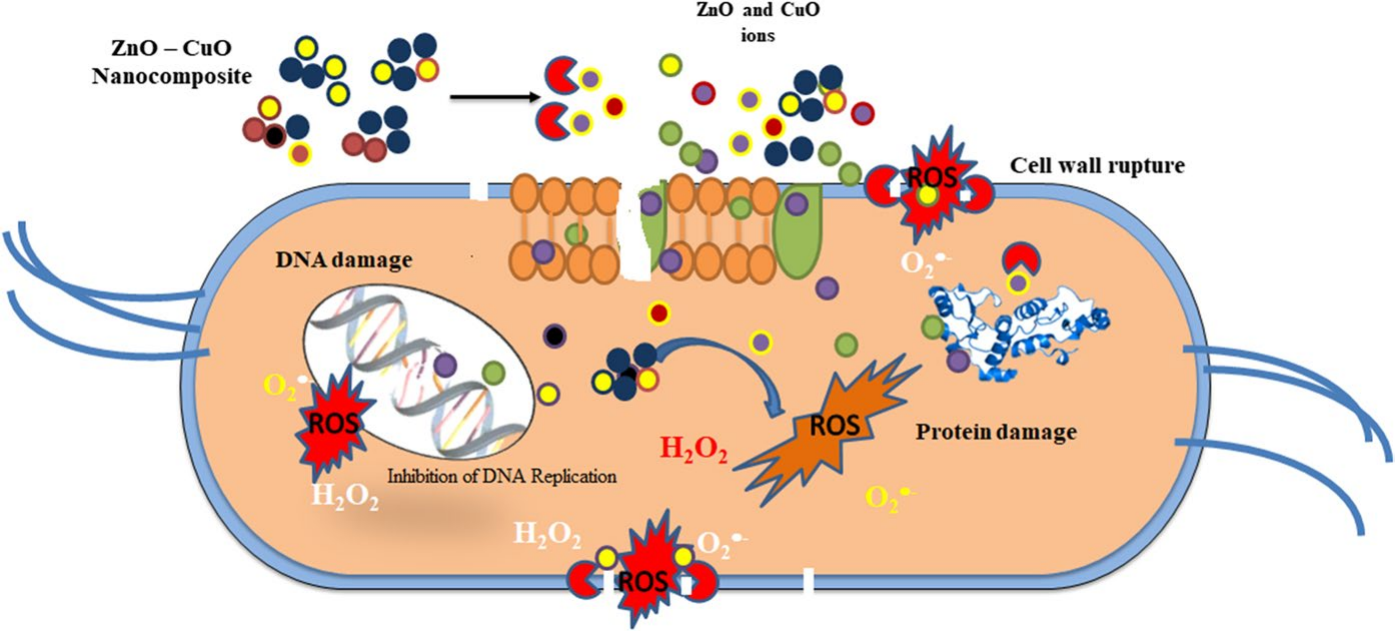
Schematic possible mechanism of antibacterial activity

**Fig. 8:**
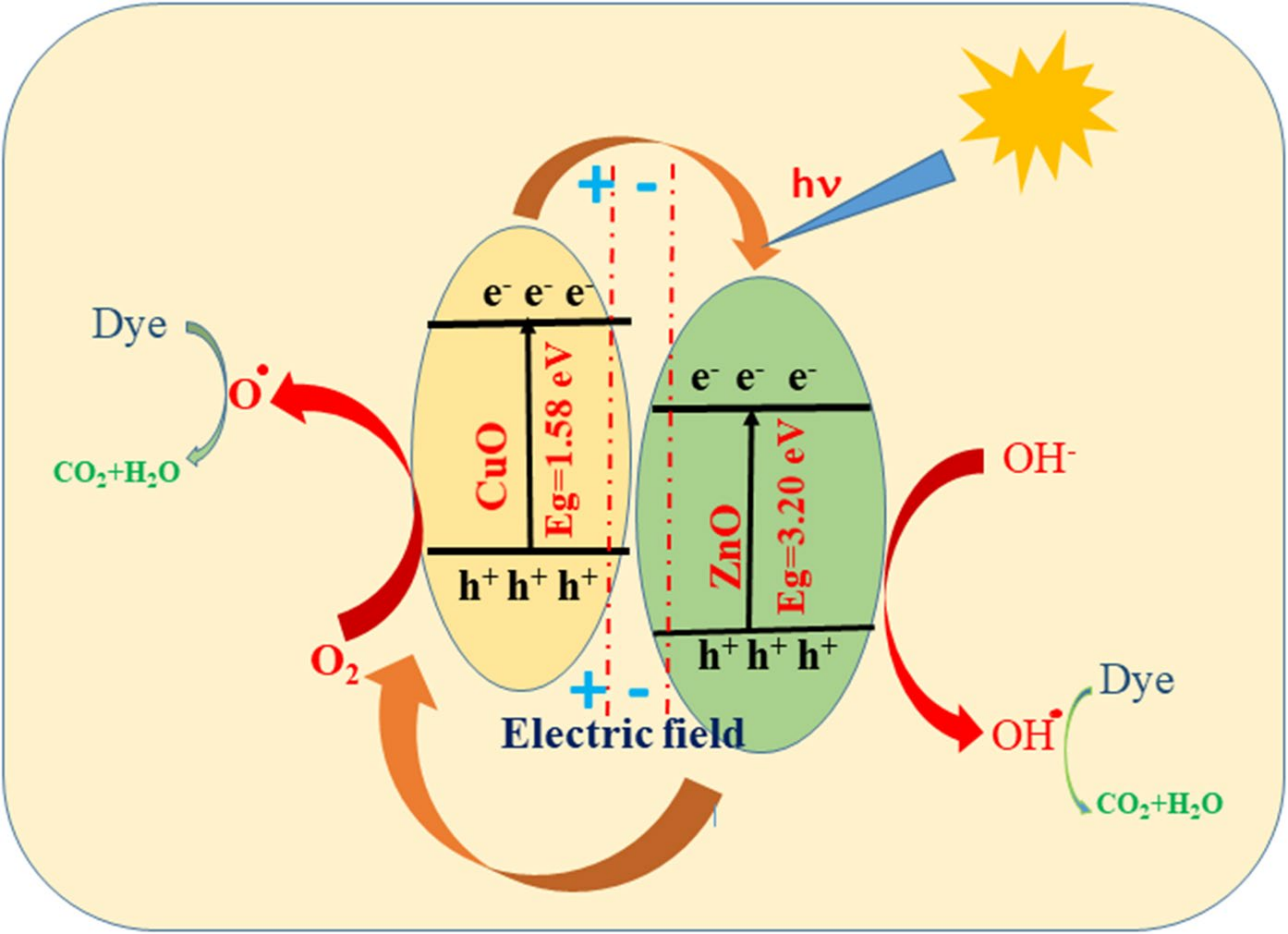
Schematic possible mechanism of dye degradation

Antimicrobial and photocatalytic activities are inclined by limited basic factors like size, surface area, optical absorption, and morphology and separation performance of photo-generated charge carriers (Aizamddin et al., 2022; Murugan et al., 2016; Saravanan et al., 2013). The antibacterial activity is depending upon the reactive oxygen species (ROS), surface area, and size of the element. Figure 7 represents the antibacterial mechanism of synthesized ZnO–CuO nanocomposite. Nanoparticles and nanocomposite can act as photocatalysts and produce ROS such as ^•^O_2_^−^,·OH, and H_2_O_2_ in the existence of dissolved O_2_ are important to extra free radical creation (Saleh & Gupta, 2011). The ZnO–CuO nanocomposite generate ROS thoroughly Fenton reaction leading to DNA damage, protein denaturation, finally it can reason for the death of the microorganism. This reaction is examined the^**.**^OH, and H_2_O_2_are dependable species for the photodegradation activity of MB dye molecule (Fig. 8; Eqs. (2)–(10)). Photocatalytic redox response by and large happens on the outside of impetuses and the upgraded surface properties significantly impact the proficiency of the photocatalyst (Arunadevi et al., 2018; Rangayasami et al., 2021).

The photocatalytic mechanism of MB over ZnO–CuO nanocomposites is illustrated in Fig. 8. It is essential to highlight that the photocatalytic reaction takes place on the surface of the photocatalyst. Heterogeneous photocatalysis by semiconductors requires fulfilment of two fundamental conditions: thermodynamic and kinetic. The formation of a heterojunction between CuO and ZnO facilitates the separation of photogenerated carriers (Klinbumrung et al., 2014). The energy band gap of the CuO and ZnO are 1.58 and 3.20 eV, respectively. In the presence of visible irradiation, CuO and ZnO are excited, resulting in the generation of electrons in the conduction band (CB) and holes in the valence band (VB), respectively, as depicted in Fig. 8. The band positions of the ZnO are below the CB and VB of CuO. The photoexcited e^−1^ transfer from CuO to ZnO, while the h^+^ migrate from ZnO to CuO. Subsequently, O_2_ molecules in the pollutant react with e^−1^ to produce superoxide radicals (·O_2_^−^), and the holes combine with H_2_O to generate hydroxyl radicals (·OH). Moreover, MB undergoes direct oxidation by the holes located at the valence band (VB) of CuO. The robust oxidant radicals of ·OH and ·O_2_^−^ efficiently oxidize the MB molecule. We suggest the following plausible degradation mechanism for MB in the presence of sunlight using CuO/ZnO (Fouda et al., 2020b; Satdeve et al., 2019).

2 $${\text{ZnO}}\left( {{\text{e}}^{ - } + \,{\text{h}}^{ + } } \right)/{\text{CuO}}\left( {{\text{e}}^{ - } + \,{\text{h}}^{ + } } \right) \, + {\text{h}}\nu \to {\text{ZnO}}\left( {{\text{e}}^{ - } + \,{\text{e}}^{ - } } \right)/{\text{CuO}}\left( {{\text{h}}^{ + } + \,{\text{h}}^{ + } } \right)$$

3 $${\text{h}}^{ + } \left( {{\text{VB}}} \right) \, + {\text{H}}_{{2}} {\text{O}} \to ^{ \cdot } {\text{OH }} + {\text{ H}}^{ + }$$

4 $${\text{e}}^{ - } \left( {{\text{CB}}} \right) \, + {\text{ O}}_{{2}} \to ^{ \cdot } {\text{O}}_{{2}}$$

5 $${\text{H}}_{{2}} {\text{O}} + ^{ \cdot } {\text{O}}_{{2}}^{ - } \to {\text{HO}}_{{2}}^{ \cdot } + {\text{OH}}^{ - }$$

6 $${\text{2HO}}_{{2}}^{ \cdot } + {\text{ H}}^{ + } + \,{\text{e}}^{ - } \to {\text{H}}_{{2}} {\text{O}}_{{2}}$$

7 $${2} ^{ \cdot } {\text{OOH}} \to {\text{O}}_{{2}} + {\text{H}}_{{2}} {\text{O}}_{{2}}$$

8 $${\text{H}}_{{2}} {\text{O}}_{{2}} + ^{ \cdot } {\text{O}}_{{2}}^{ - } \to ^{ \cdot } {\text{OH}} + {\text{OH}}^{ - } + {\text{O}}_{{2}}$$

9 $$^{ \cdot } {\text{OH}} + ^{ \cdot } {\text{O}}_{{2}}^{ - } + {\text{h}}^{ + } \left( {{\text{VB}}} \right) + {\text{pollutants}} \to {\text{degrade pollutant}}$$

10 $$^{ \cdot } {\text{OH}} + ^{ \cdot } {\text{O}}_{{2}}^{ - } + {\text{h}}^{ + } \left( {{\text{VB}}} \right) + {\text{degrade pollutant}} \to {\text{CO}}_{{2}} \uparrow + {\text{H}}_{{2}} {\text{O}}$$

## Conclusion

A novel ZnO–CuO nanocomposite in *Carica papaya* leaf extract was successfully synthesized through eco-friendlymethod. Leaf extract of *Carica papaya* extract have successfully mediated their composite due to stabilizing agent of phytochemical present in extract. The crystalline structure of nanocomposite was revealed using XRD analysis. The cubic like structure was determined by SEM analysis. Further, the ZnO–CuO photocatalyst shows outstanding photocatalytic efficiency of MB under visible light irradiation. Thee maximum degradation efficiency of 96.73% was achieved within 120 min. Moreover. *Carica papaya* plant extract stabilized ZnO–CuO nanocomposite shows better antibacterial agent against E. coli, S. aureus, K. pneumonia and B. subtilis. Furthermore, with the advantage of utilizing Carica papaya leaf extract to stabilize ZnO–CuO composites, the synthesis becomes environmentally friendly and cost-effective. The resulting flexible heterojunction ZnO–CuO composites offer promising prospects for applications such as adsorbents, electrochemical materials, and sensor support.
